# Risk of aortic aneurysm and aortic dissection with the use of fluoroquinolones in Korea: a nested case–control study

**DOI:** 10.1186/s12872-022-02488-x

**Published:** 2022-02-13

**Authors:** Nayeong Son, Eunmi Choi, Soo Youn Chung, Soon Young Han, Bonggi Kim

**Affiliations:** Korea Institute of Drug Safety and Risk Management, 6th FL, 30, Burim-ro 169beon-gil, Dongan-gu, Anyang-si, Gyeonggi-do Republic of Korea

**Keywords:** Fluoroquinolone, Aortic aneurysm, Aortic dissection, Drug safety, Pharmacovigilance, Adverse effect

## Abstract

**Background:**

Recent studies have raised concern about the association of fluoroquinolones with an increased risk of aortic aneurysm and aortic dissection. We aimed to evaluate such risk in a Korean population.

**Methods:**

We conducted a nested case–control study using data from the National Health Insurance Service collected from 2013 to 2017 in Korea. The study cohort included patients older than 40 years and excluded patients who had used fluoroquinolones or been diagnosed with aortic aneurysm, aortic dissection, or related diseases 1 year prior to the cohort entry date. We randomly matched four controls in the risk set with each case of aortic aneurysm and aortic dissection (same sex, age, and cohort entry date). We assessed the risk of aortic aneurysm and aortic dissection from fluoroquinolones and adjusted for potential confounders using a conditional logistic regression model.

**Results:**

A total of 29,638 aortic aneurysm and aortic dissection patients were identified between 2014 and 2017. The use of fluoroquinolones within a year was associated with a 10% increased risk of aortic aneurysm and aortic dissection (adjusted odds ratio: 1.10, 95% CI 1.07–1.14, *p* < 0.05) compared with nonusers. The risk was higher in patients who had used fluoroquinolones within 60 days (adjusted odds ratio: 1.53, 95% CI 1.46–1.62, *p* < 0.05). The risk of aortic aneurysm and aortic dissection positively correlated with the cumulative dose and duration of fluoroquinolone therapy (*p* < 0.001).

**Conclusions:**

Our study provides real-world evidence of the risk of aortic aneurysm and aortic dissection from fluoroquinolones in Korea. Patients and medical professionals should be aware that fluoroquinolones can increase the risk of aortic aneurysm and aortic dissection, which may be acerbated by high dosage and duration of use.

**Supplementary Information:**

The online version contains supplementary material available at 10.1186/s12872-022-02488-x.

## Background

Fluoroquinolones (FQs) are among the most widely used antibiotics in Korea, and their use has consistently increased to account for 9 to 11% of all antibiotic use [1]. Although FQs are powerful antibiotics with a wide antibacterial spectrum [2], they induce degradation of collagen and other structural components of the extracellular matrix by stimulating matrix metalloproteinases [3]. The possibility of excessive tissue breakdown by this mechanism has raised concern about the risk of adverse reactions, such as aortic aneurysm (AA) and aortic dissection (AD).

In December 2018, the U.S. Food and Drug Administration warned that FQs can increase the occurrence of rare but serious ruptures or tears in the aorta. The warning included special caution for patients with a history of aneurysms, blockages, or hardening of the arteries, high blood pressure, or genetic conditions such as Marfan or Ehlers–Danlos syndrome and instructed patients to inform their health-care professional before starting a fluoroquinolone prescription [4]. Following that warning, the Ministry of Food and Drug Safety in Korea issued a safety letter warning about the potential association of fluoroquinolone use and the risk of AA/AD [5].

Many observational studies have suggested that fluoroquinolone use could be significantly associated with an increased risk of AA/AD [3, 6–9]. Recently, a systemic review and meta-analysis showed that fluoroquinolone use incurs a risk of developing three collagen-associated diseases, including AA/AD [10]. However, it has not yet been established whether fluoroquinolone use increases the risk of AA/AD in the Korean population. This study aims to evaluate the association between fluoroquinolone use and the risk of AA/AD in the Korean population.

## Methods

### Data source

We conducted a nested case-control study using National Health Insurance Service (NHIS)-customized data (NHIS-2019-1-024). The NHIS database covers almost 98% of the total population in Korea. It contains patient demographic information such as sex, date of birth, date of death, and medical treatment records, including details of disease and prescriptions [11]. The authors declare no conflicts of interest with NHIS.

### Study population

The study population comprised all patients aged 40 to 99 years 2014–2017 in the NHIS database. The date of 1 January 2014 was defined as the cohort entry date for patients aged 40 years or older in 2014. For patients aged less than 40 years in 2014, we established the cohort entry date as the first day of the year that the patient became 40 years old. We excluded 510,805 patients who:

- Had taken FQs more than once during the year prior to the cohort entry date
- Were diagnosed with AA/AD during the year prior to the cohort entry date
- Were diagnosed with underlying related diseases (atherosclerosis of the aorta, arteritis, aortitis, Lerche’s syndrome, coarctation of the aorta, Marfan’s syndrome, valve diseases, endocarditis, congenital malformations of valves, heart failure) (Additional file 1: Table S1) during the year prior to the cohort entry date.

#### Case selection

From the cohort, we identified 29,638 patients aged 40 years or older who had experienced AA/AD from 2014 to 2017 according to the definition of health outcomes of interest “Statistical analysis” section . Patients in the case group were observed from the cohort entry date to the index date, which was defined as the first date of diagnosis of AA/AD.

#### Control selection

After we stratified the case group based on age and sex, we created a risk set for each case using patients who were of the same sex and age as those with AA/AD and did not have a history of an AA/AD diagnosis. The size of the risk set was 20 times the sample size of each stratum. We randomly matched four controls in the risk set. Patients in the control group were observed from the cohort entry date to the index date of matched cases.

### Health outcomes of interest

The outcome of the main analysis was defined as a diagnosis of AA/AD after entry to the cohort. Incident cases were defined as those who had received an ICD 10 code I71 (ICD 10 I71.0–I71.9) for all kinds of AA/AD. The outcome for the sensitivity analysis was redefined as a diagnosis of AA/AD in addition to having received a laboratory test specific for AA/AD (abdominal/thoracic aortography, computed tomography (CT), magnetic resonance imaging, ultrasonography, Doppler echocardiography, transesophageal/transthoracic echocardiography, abdominal vascular ultrasonography, or aorta Doppler ultrasonography) within 28 days prior to the diagnosis. The diagnosis and treatment of AA/AD were based on the ACCF/AHA/AATS/ACR/ASA/SCA/SCAI/SIR/STS/SVM guideline in the general Korean hospitals [12]. The first date of diagnosis was defined as the index date for cases and matched controls.

### Exposure

The exposure of interest was the use of a fluoroquinolone (balofloxacin, ciprofloxacin, enoxacin, gatifloxacin, gemifloxacin, levofloxacin, lomefloxacin, moxifloxacin, norfloxacin, ofloxacin, tosufloxacin, and zabofloxacin) in the year prior to the index date.

We categorized fluoroquinolone users as current, recent, or past users according to the time from the end of supply of the fluoroquinolone prescription to the index date. In this definition, ‘termination of fluoroquinolone exposure’ means the end of supply of the fluoroquinolone prescription. Current users were defined as patients who had terminated fluoroquinolone exposure within the 60 days prior to the index date. Recent users were defined as patients who had terminated fluoroquinolone exposure 61–120 days prior to the index date. Past users were defined as patients who had terminated fluoroquinolone exposure 121–365 days prior to the index date.

To investigate the effects of cumulative dose and duration of FQ exposure on the prevalence of AA/AD, we categorized fluoroquinolone users into three groups according to the quantiles of duration and into four groups according to the quantiles of cumulative dose. The duration was calculated as the sum of the total days of supply for each prescription in the year prior to the index date. The first and third quantiles of the cumulative days of supply were found to be 2 and 14 days, respectively. We represented the cumulative dose in the year prior to the index date in terms of the defined daily dose (DDD), as defined by the anatomical therapeutic chemical classification system. The first, second, and third quantiles of cumulative dose were found to be 4 DDD, 7.5 DDD, and 15 DDD, respectively.

The NHIS dataset included the Korean ingredient code of the drug, the date the prescription was written, the number of days of supply, and the quantity. We used these data to identify prescriptions for FQs and any concomitantly used drugs.

### Statistical analysis

Pearson Chi-square tests and Fisher’s exact test were used for the analysis of categorical variables. The odds ratios of the association between FQ use and AA/AD were calculated using multivariate conditional logistic regression analysis. We considered covariates known to be related to AA/AD or fluoroquinolone use from previous studies and included them as confounders in the model [3, 6–9]. The covariates are listed in Table 1. We also tested the tendency of AA/AD to occur with changes in timing, cumulative dose, and duration of FQ use using the Cochran-Armitage trend test. All data processing and statistical analyses were performed using SAS 9.4 and R 5.3.1 using two-sided tests, and a *p* value of < 0.05 was considered significant.

**Table 1:** Demographics and clinical characteristics of the study population

	Case (29,638)		Control (118,552)		*p* value*
	N	(%)	N	(%)	
**Sex**					
Male	18,529	62.5	74,116	62.5	1
Female	11,109	37.5	44,436	37.5	
**Age (year)**					
40–49	2080	7	8320	7	1
50–59	4422	14.9	17,688	14.9	
60–69	7031	23.7	28,124	23.7	
70–79	9479	32	37,916	32	
80–89	5748	19.4	22,992	19.4	
90 +	878	3	3512	3	
**Underlying disease**					
Cerebrovascular disease	3124	10.5	6418	5.4	< 0.001
Arterial disease	8227	27.8	22,080	18.6	< 0.001
Ischemic heart disease	7845	26.5	14,715	12.4	< 0.001
Cardiac valve disease	884	3	444	0.4	< 0.001
Conduction disorder	115	0.4	272	0.2	< 0.001
Heart failure or cardiomyopathy	2723	9.2	4108	3.5	< 0.001
Chronic obstructive pulmonary disease	10,626	35.9	31,726	26.8	< 0.001
Pneumonia	3579	12.1	9047	7.6	< 0.001
Cancer	3422	11.5	8462	7.1	< 0.001
Liver disease	10,520	35.5	32,012	27	< 0.001
Renal disease	1773	6	2902	2.4	< 0.001
Rheumatism	2005	6.8	5720	4.8	< 0.001
Psychiatric disorder	13,498	45.5	41,650	35.1	< 0.001
Diabetes	9518	32.1	33,700	28.4	< 0.001
Hypertension	19,716	66.5	60,520	51	< 0.001
Lipid disorder	16,673	56.3	50,268	42.4	< 0.001
Trauma	15,191	51.3	53,280	44.9	< 0.001
Obstructive sleep apnea	73	0.2	190	0.2	0.002
Asthma	6747	22.8	19,825	16.7	< 0.001
Obesity	41	0.1	116	0.1	0.069
Seizure disorder	1225	4.1	3015	2.5	< 0.001
Decubitus ulcer	9	0	26	0	0.526
Infectious disease	13,949	47.1	46,881	39.5	< 0.001
Hypothyrodism	1729	5.8	5087	4.3	< 0.001
Inflammatory bowel disease	3876	13.1	11,773	9.9	< 0.001
Urinary tract infection	2287	7.7	5442	4.6	< 0.001
Ehlers–Danlos syndrome	1	0	0	0	0.2
**Charlson comorbidity Index**					
Mean(SD)	2.67(2.41)	1.86(2.07)			
0	5371	18.1	37,377	31.5	< 0.001
1	5781	19.5	27,124	22.9	
2	5425	18.3	19,785	16.7	
3+	13,061	44.1	34,266	28.9	
Myocardial infarction	1377	4.6	1924	1.6	< 0.001
Congestive heart failure	3731	12.6	6315	5.3	< 0.001
Peripheral vascular disease	7376	24.9	20,782	17.5	< 0.001
Cerebrovascular disease	6913	23.3	16,487	13.9	< 0.001
Dementia	3995	13.5	11,955	10.1	< 0.001
Chronic pulmonary diseases	13,066	44.1	40,316	34	< 0.001
Connective tissue disease	1827	6.2	5172	4.4	< 0.001
Peptic ulcer	10,817	36.5	33,712	28.4	< 0.001
Mild liver diseases	9697	32.7	29,509	24.9	< 0.001
Uncomplicated diabetes	106	0.4	427	0.4	0.991
Diabetes complicated with retinopathy, neuropathy, renal disease	2868	9.7	10,911	9.2	0.012
Hemiplegia	936	3.2	2016	1.7	< 0.001
Moderate or severe renal diseases	1773	6	2902	2.4	< 0.001
Nonmetastatic solid cancer, leukemia, lymphoma, multiple myeloma	3288	11.1	8082	6.8	< 0.001
Moderate or severe liver diseases	180	0.6	545	0.5	0.001
Metastatic solid cancer	337	1.1	765	0.6	< 0.001
AIDS/HIV	7	0	25	0	0.965
Medication use**					
Angiotensin-converting enzyme inhibitors	1119	3.8	2174	1.8	< 0.001
Antiarrhythmic	13,410	45.2	34,164	28.8	< 0.001
Anticonvulsant	3375	11.4	8889	7.5	< 0.001
Antidepressant	5542	18.7	14,066	11.9	< 0.001
Immunodepressant	8901	30	27,220	23	< 0.001
Anticoagulant	20,898	70.5	68,727	58	< 0.001
β-blocker	7664	25.9	15,373	13	< 0.001
Oral hypoglycemic agent	3935	13.3	18,862	15.9	< 0.001
Benzodiazepine	10,934	36.9	30,683	25.9	< 0.001
Calcium Channel Blockers	13,194	44.5	38,216	32.2	< 0.001
corticosteroid	16,632	56.1	56,174	47.4	< 0.001
Disease-modifying antirheumatic drugs	618	2.1	1559	1.3	< 0.001
Insulin	1136	3.8	2876	2.4	< 0.001
Loop diuretics	3483	11.8	5350	4.5	< 0.001
Nonsteroidal anti-inflammatory drugs	23,354	78.8	82,504	69.6	< 0.001
Antipsychotic	7588	25.6	23,261	19.6	< 0.001
Peripheral vasodilators	3425	11.6	4115	3.5	< 0.001
Lipid-lowering agent	11,079	37.4	31,002	26.2	< 0.001
Parkinson medication	1990	6.7	6220	5.2	< 0.001
Hydroxyzine	2616	8.8	8049	6.8	< 0.001
Cardiac or aortic procedure/surgery	287	1	251	0.2	< 0.001

*The *p* values are results from Chi-square or Fisher’s exact tests

**Information on underlying disease were derived from data recorded prior to the index date and after cohort entry. Information on medication use were derived from data recorded in 1 year prior to the index date

## Results

### Demographic and clinical characteristics

The final study population was composed of 148,190 patients, including 29,638 cases and 118,552 controls. Table 1 shows the baseline characteristics of the study population. This cohort comprised 92,645 male patients (62.5%) and 55,545 female patients (37.5%). More than half of the study population was 60–69 years old (23.7%) or 70–79 years old (32.0%). Patients in the AA/AD case group had a higher prevalence of cerebrovascular disease and cardiovascular disorders such as arterial disease and ischemic heart disease. In the year prior to the index date. Patients in the AA/AD case group were more often users of angiotensin-converting enzyme inhibitors, antiarrhythmics, anticonvulsants, etc. from the cohort entry date to the index date and experienced more cardiac or aortic procedures and surgeries in the previous year.

### Association between AA/AD and FQ use

**Fig. 1:**
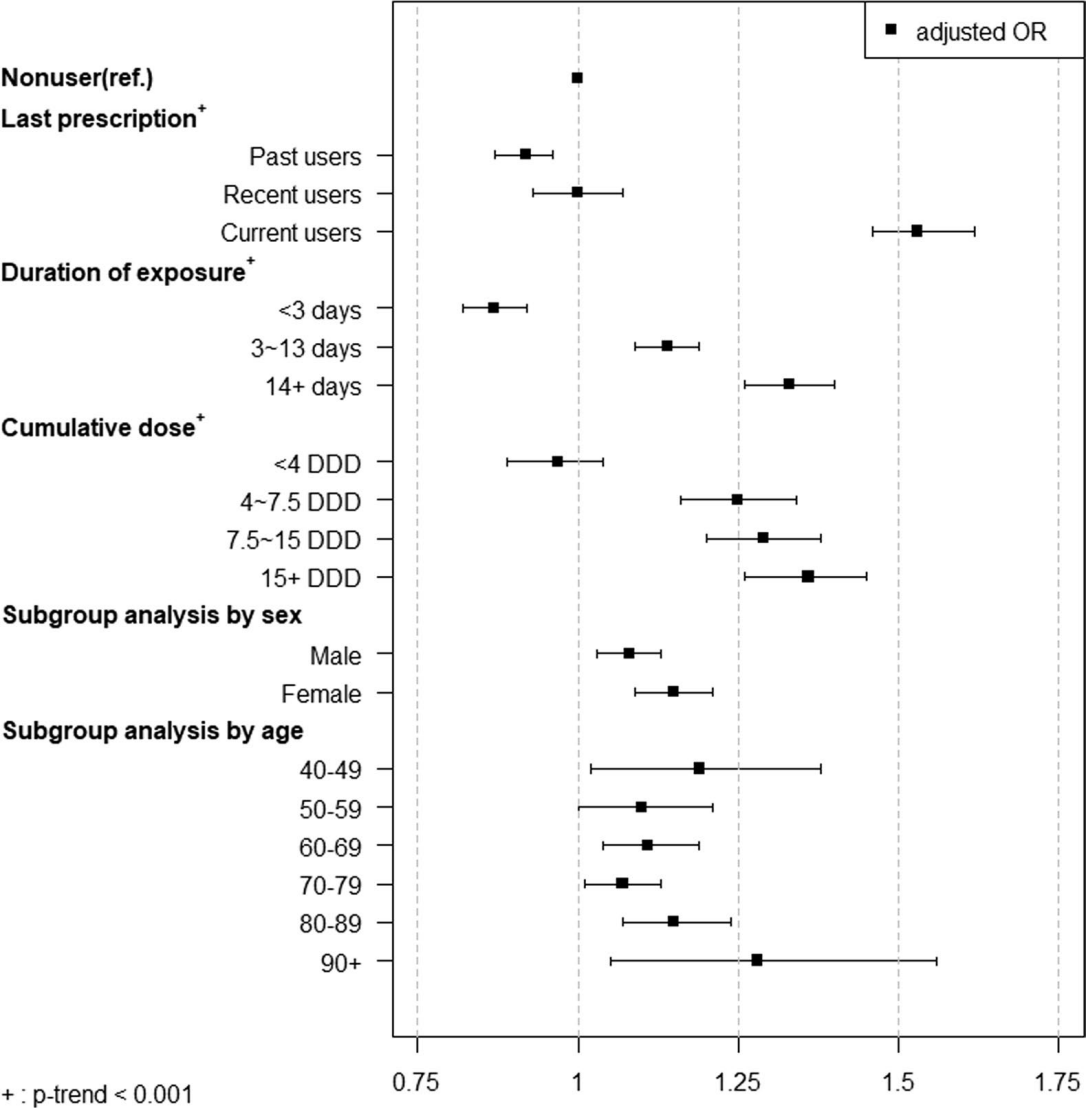
Results of conditional logistic regression analysis of the association between AA/AD and FQ use

During the observation period (1 year before the index date), 8562 cases (28.9%) and 25,387 controls (21.4%) received at least one prescription for FQs. Table 2 and Figure 1 show the results of the conditional logistic regression analysis. The adjusted odds ratio was 1.10 (95% CI 1.07–1.14, *p* < 0.05) during the 1-year observation period. However, the risk was substantially higher in current users (adjusted OR 1.53, 95% CI 1.46–1.62, *p* < 0.05). FQ use did not have a significant association with AA/AD in recent users (adjusted OR 1.00, 95% CI 0.93–1.07, *p* < 0.05). The risk was even lower in past users (adjusted OR 0.92, 95% CI 0.87–0.96, *p* < 0.05).

**Table 2:** Results of conditional logistic regression analysis of the association between AA/AD and FQ use

	Case		Control		Crude OR		Adjusted OR*	
	N	%	N	%	OR	95% CI	OR	95% CI
**Main analysis**								
Nonusers	21,076	71.1	93,165	78.6	1	–	1	–
Users	8562	28.9	25,387	21.4	1.51**	1.47–1.56	1.10**	1.07–1.14

*Adjusted for covariates presented in Table 1 (sex, age, underlying disease, Charlson comorbidity index, medication use, history of procedure/surgery)

**p < 0.05

The risk of AA/AD was studied according to the duration of exposure and the cumulative dose of FQs. In this study, 25% of FQ users were exposed to FQs for 2 days or less. On the other hand, 25% of the FQ users were exposed to FQs for more than 14 days. Among them, 50% of the FQ users were exposed to FQs for between 3 days and 13 days. We used the same covariates as those adopted for the primary analysis. Patients who used FQs for less than three days had a lower risk of AA/AD than nonusers (adjusted OR 0.87, 95% CI 0.82–0.92, *p* < 0.05). However, the risk was significantly higher in patients who had used FQs for between three days and 13 days (adjusted OR 1.14, 95% CI 1.09–1.19, *p* < 0.05) and was highest in patients who used FQs for more than 14 days (adjusted OR 1.33, 95% CI 1.26–1.40, *p* < 0.05).

FQ users were categorized into four groups with regard to dose (low, mid-low, mid-high, or high) according to the quantiles of cumulative dose. Patients in the low-dose group had used FQs less than 4 DDDs during the observation period. Patients in the mid-low dose group and mid-high dose group had used 4 DDDs to 7.5 DDDs and 7.5 DDDs to 15 DDDs, respectively. Patients in the high-dose group had used more than 15 DDDs during the observation period. Compared with nonusers, the risk of AA/AD in the low-dose group (<4 DDDs) was not significantly higher (adjusted OR 0.97, 95% CI 0.89–1.04, *p* > 0.05). However, the risk was significantly higher in patients who had used FQs at more than 4 DDDs. Specifically, the adjusted odds ratio of AA/AD was 1.25 (95% CI 1.16–1.34, *p* < 0.05) in the mid-low dose group, 1.29 (95% CI 1.20–1.38, *p* < 0.05) in the mid-high dose group, and 1.36 (95% CI 1.26–1.45) in the high dose group.

### Subgroup analysis and sensitivity analysis

From the subgroup analysis by sex (Fig. 1), we found that the association between AA/AD and FQ use remained statistically significant in both the male and female subgroups. In particular, the risk was high in female patients (adjusted OR 1.15, 95% CI 1.09–1.21, *p* < 0.05) compared with female nonusers. When ages were grouped into 10-year bands, the association between AA/AD and FQ use remained statistically significant in every age group.

To verify the consistency of the results, we performed sensitivity analysis (Table 3) by changing the definition of AA/AD occurrence. The AA/AD occurrence in the primary analysis was identified using the ICD 10 code for AA/AD. For the sensitivity analysis, we changed the definition of an AA/AD case to a diagnosis of AA/AD in addition to having received a laboratory test specific for AA/AD within the 28 days prior to the initial diagnosis of AA/AD. Among 29,648 AA/AD cases, 21,528 (72.6%) received the laboratory test specific for AA/AD within the 28 days prior to the initial diagnosis of AA/AD. Among those 21,528 patients, 17,875 (83.0%) were diagnosed with AA/AD the day they took the tests. Abdominal/thoracic CT, aortography, and transthoracic echocardiography were found to have been the commonly performed procedures. The results remained consistent with the primary results under the new definition.

**Table 3:** Results of sensitivity analysis of the association between AA/AD and FQ use

	Case		Controls		Crude OR		Adjusted OR*	
	N	%	N	%	OR	95% CI	OR	95% CI
**Sensitivity analysis**								
Nonusers	15,294	71.0	67,570	78.5	1	–	1	–
Users	6234	29.0	18,542	21.5	1.51**	1.46–1.56	1.10**	1.06–1.14

Cases that received laboratory tests specific for AA/AD within 28 days prior to the initial diagnosis of AA/AD and their matched controls were included

*Adjusted for covariates presented in Table 1 (sex, age, underlying disease, Charlson comorbidity index, medication use, history of procedure/surgery)

**p < 0.05

The risk of AA/AD by FQs was substantially higher in current users. The risk increased as the duration of exposure and cumulative dose increased. The association remained statistically significant in every subgroup by sex and age. See Additional file 1: Table S2 for numeric results.

## Discussion

In this study, FQ use showed a trend to be associated with an increased risk of AA/AD during the 1-year observation period, but the effect size was not remarkable. However, the risk of AA/AD in current users of FQs was relatively considerable. This result is in line with preceding research in many ways. In an in vitro study that assessed the effect of FQs on MMP activities in human aortic smooth muscle cells, 48 hours of treatment with ciprofloxacin significantly increased total MMP activity. Observational studies using Taiwanese and Swedish databases also showed that the risk of AA/AD within 60 days after FQ use was significantly higher than that of nonusers [3, 7, 8]. In addition, a cohort study in Ontario, Canada and a signal analysis using U.S. FAERS data also indicated significant associations between FQ use and AA/AD [6, 9]. This trend is consistent with the results of a systematic literature review and meta-analysis conducted in 2019 [10]. In particular, Pasternak et al. [3] showed that the cumulative incidence of AA/AD increased significantly during the first 10 days after FQ use. Given these findings, further studies are needed to evaluate the risk in the early period of FQ use.

Studies that utilized the Taiwanese database [7, 8] reported that the risk of AA/AD increased as the duration of drug use increased. In this study, the adjusted odds ratio of AA/AD also increased as the cumulative duration of FQ use increased. In addition, while no prior study has determined the effect of the cumulative dose of FQs on the risk for AA/AD, this study showed that an increased cumulative dose of FQs could increase the risk for AA/AD. The dose–response relationship and duration-response relationship can be interpreted as considerable evidence of the causal relationship between FQ use and the occurrence of AA/AD. Therefore, the patient's condition should be carefully monitored, keeping in mind that the risk of AA/AD may increase as the cumulative dose or duration of FQ use increases.

Our study suggests some different results from the general understanding of AA/AD. In general, AA/AD progresses slowly over several years, and men and old age are known as risk factors. However, we found that the risk of AA/AD from FQ use was significant (1) in the early period of FQ use, (2) in female patients and male patients, and (3) in all age groups. In this research, the risk of AA/AD was 8% higher in male FQ users and 15% higher in female FQ users than in nonusers of each sex. Although the risk difference between female patients and male patients was not statistically significant, it gives us a reasonable inference that female patients may have a higher risk of FQ-induced AA/AD, contrary to general knowledge that the incidence of AA/AD is higher in male patients. A previous study also showed that the risk was higher in female patients [7]. For age, the risk was significant in all age groups, but the differences between subgroups were not statistically significant. Given that the risk was higher in patients aged 70 or older in a previous study [7], we recommend that further research be undertaken to understand the risk factors for FQ-induced AA/AD. The sensitivity analysis supported the robustness of the results, as they were very similar to the results before the definition of the study population was changed.

### Strengths and limitations

As an indication of the strength of this research, it was conducted using the national health insurance claim data of all adults aged 40 or older in Korea during the five years from 2013 to 2017. The NHIS-customized data are well accumulated in the form of detailed medical activities and drugs, making it easy to generalize the research results, as nearly all domestic AA/AD patients were included in the study population. Additionally, we comprehensively considered various confounding factors, such as underlying diseases, medication use, and procedures and surgeries related to AA/AD. Moreover, we performed a sensitivity analysis by changing the definition of health outcomes of interest to minimize the effect of classification errors on the results. The preceding research results showed a 92% positive prediction for the identification of AA/AD cases when defining a group of cases considering both examination and diagnosis [7].

Our work clearly has some limitations. First, the results may have been affected by confounding indications. To reduce the bias from confounding indications, we excluded patients who had taken FQs during the year prior to the cohort entry date and included major indications of FQs as covariates in the adjusted model. However, the results may still have been affected by unmeasured underlying indications or the severity of the indication. The result must be carefully interpreted considering that patients who take FQs are possibly at higher risk of AA/AD due to unmeasured underlying conditions, indications for the drug, and important risk factors such as smoking. We emphasize that this result should not be interpreted as explicit evidence for causal effects. It is clear that more studies would be necessary to determine whether there is a causal relationship.

Second, due to the nature of the claim data, it is difficult to pinpoint the exact timing of treatment and drug use, and it is not possible to analyze drug use, procedures, or surgeries that are not covered by NHIS. Third, socioeconomic and clinical confounding factors that could not be measured or predicted may have affected the results. For example, the difference in baseline characteristics of cases and controls can affect the results. To minimize the effect of known risk factors for AA/AD, we excluded patients with a history of AA/AD or related diseases during the year prior to the cohort entry date. However, some risk factors generally known to affect AA/AD, such as blood pressure, smoking status, and family history, were not considered in this study. In this sense, further studies are needed to evaluate the risk by patients’ baseline health status and particular medical conditions, such as known risk factors for AA/AD.

Finally, the various clinical types and characteristics of AA/AD were not analyzed because clinical information such as severity and detailed disease symptoms could not be fully determined by the diagnosis code alone. Thus, the results of this study are not appropriate for direct application to individuals, as patients may present with a variety of clinical characteristics. To overcome the potential bias introduced by confounding factors and the definition of exposure and health outcome of interest, we performed subgroup analysis, sensitivity analysis, and examined the dose–response relationship.

## Conclusion

In this nested case–control study, we found that the use of FQs within a year was associated with a 10% increased risk of AA/AD in the Korean population. AA/AD is a life-threatening disease accompanied by severe complications such as low blood pressure, shock, myocardial infarction, stroke, lower limb paralysis, and acute renal failure, which can lead to sudden death. In particular, early diagnosis and prompt treatment of abdominal AA/AD are critical, as 65% of patients die from cases of rupture [13]. Therefore, if patients feel symptoms such as chest pain in the early period of FQ use, even if the patient is not in the previously known risk group, medical professionals should suspect acute FQ-induced AA/AD, make a close diagnosis and consider changing or stopping the prescription. Moreover, if FQs are used in patients with already identified AA/AD, medical professionals should review the patient's history and carefully monitor them after drug administration, keeping in mind that FQs could increase the risk of AA/AD and that the cumulative dose or duration of FQ use may affect the risk.

## Supplementary Information

**Additional file 1: Table S1.** ICD 10 code of AA/AD–related disease. **Table S2.** Results of conditional logistic regression analysis of the association between AA/AD and FQ use. **Table S3.** Frequency of underlying disease and mediation use in exposed and unexposed controls. **Table S4.** Association between AA/AD and FQ use in patients with cardiovascular diseases or indications of FQs.

## Data Availability

The data that support the findings of this study are available from the National Health Insurance Service in the Republic of Korea, but restrictions apply to the availability of these data, which were used under license for the current study and so are not publicly available. Data are, however, available from the authors upon reasonable request and with permission from the National Health Insurance Service.

## Abbreviations

AA: Aortic aneurysm
AD: Aortic dissection
DDD: Defined daily dose
FQs: Fluoroquinolones
NHIS: National Health Insurance Service
