# Supplementary material for: Risk of aortic aneurysm and aortic dissection with the use of fluoroquinolones in Korea: a nested case–control study
Source: BMC Cardiovasc Disord. 2022 Feb 13;22:44. doi: 10.1186/s12872-022-02488-x (PMC8842902; doi:10.1186/s12872-022-02488-x)
Supplement: Supplementary file 1 — Additional file 1: Table S1. ICD 10 code of AA/AD–related disease. Table S2. Results of conditional logistic regression analysis of the association between AA/AD and FQ use. Table S3. Frequency of underlying disease and mediation use in exposed and unexposed controls. Table S4. Association between AA/AD and FQ use in patients with cardiovascular diseases or indications of FQs. [file 12872_2022_2488_MOESM1_ESM.docx]

**Supplementary Table**

**Table 1. ICD 10 code of AA/AD–related disease**

| **ICD 10** | **Name** |
| --- | --- |
| I70.0 | Atherosclerosis of aorta |
| I77.6 | Arteritis, unspecified |
| I79.1 | Aortitis in diseases classified elsewhere |
| I74.0 | Embolism and thrombosis of abdominal aorta  Aortic bifurcation syndrome  Leriche syndrome |
| Q25.1 | Coarctation of aorta |
| Q87.4 | Marfan syndrome |
| I05 | Rheumatic mitral valve diseases |
| I06 | Rheumatic aortic valve diseases |
| I07 | Rheumatic tricuspid valve diseases |
| I08 | Multiple valve diseases |
| I34 | Nonrheumatic mitral valve disorders |
| I35 | Nonrheumatic aortic valve disorders |
| I36 | Nonrheumatic tricuspid valve disorders |
| I37 | Pulmonary valve disorders |
| I38 | Endocarditis, valve unspecified |
| I39.8 | Endocarditis, valve unspecified, in diseases classified elsewhere |
| Q22 | Congenital malformations of pulmonary and tricuspid valves |
| Q23 | Congenital malformations of aortic and mitral valves |
| I50 | Heart failure |

* Each code includes its subcodes.

**Table 2. Results of conditional logistic regression analysis of the association between AA/AD and FQ use**

| **Category** | | **Case** | | **Control** | | **Crude OR** | | **Adjusted OR*** | |
| --- | --- | --- | --- | --- | --- | --- | --- | --- | --- |
|  |  | **N** | **%** | **N** | **%** | **OR** | **95% CI** | **OR** | **95% CI** |
| Nonusers | | 21,076 | 71.1 | 93,165 | 78.6 | 1 | - | 1 | - |
| Last prescription^†^ | Past users | 3,737 | 12.6 | 13,920 | 11.7 | 1.22 | 1.17–1.27 | 0.92^**^ | 0.87–0.96 |
|  | Recent users | 1,454 | 4.9 | 4,713 | 4.0 | 1.37 | 1.29–1.46 | 1.00 | 0.93–1.07 |
|  | Current users | 3,371 | 11.4 | 6,754 | 5.7 | 2.22 | 2.12–2.33 | 1.53^**^ | 1.46–1.62 |
| Duration of exposure^†^ | < 3 days | 1,833 | 6.2 | 7,741 | 6.5 | 1.05 | 1.00–1.11 | 0.87^**^ | 0.82–0.92 |
|  | 3 ~ 13 days | 3,754 | 12.7 | 11,087 | 9.4 | 1.52 | 1.46–1.59 | 1.14^**^ | 1.09–1.19 |
|  | 14+ days | 2,975 | 10.0 | 6,559 | 5.5 | 2.06 | 1.96–2.16 | 1.33^**^ | 1.26–1.40 |
| Cumulative dose^†^ | < 4 DDD | 1,096 | 4.1 | 3,186 | 3.3 | 1.37 | 1.27–1.47 | 0.97 | 0.89–1.04 |
|  | 4 ~ 7.5 DDD | 1,351 | 5.1 | 3,169 | 3.3 | 1.72 | 1.61–1.84 | 1.25^**^ | 1.16–1.34 |
|  | 7.5 ~ 15 DDD | 1,459 | 5.5 | 3,097 | 3.2 | 1.90 | 1.78–2.03 | 1.29^**^ | 1.20–1.38 |
|  | 15+ DDD | 1,717 | 6.4 | 3,081 | 3.2 | 2.26 | 2.13–2.41 | 1.36^**^ | 1.26–1.45 |
| **Subgroup analysis, by sex** | | | | | | | | | |
| Male | Nonusers | 13,420 | 72.4 | 58,925 | 79.5 | 1 | - | 1 | - |
|  | Users | 5,109 | 27.6 | 15,191 | 20.5 | 1.50 | 1.45–1.56 | 1.08^**^ | 1.03–1.13 |
| Female | Nonusers | 7,656 | 68.9 | 34,240 | 77.1 | 1 | - | 1 | - |
|  | Users | 3,453 | 31.1 | 10,196 | 22.9 | 1.54 | 1.47–1.61 | 1.15^**^ | 1.09–1.21 |
| **Subgroup analysis, by age** | | | | | | | | | |
| 40~49 | Nonusers | 1,684 | 81.0 | 7,246 | 87.1 | 1 | - | 1 | - |
|  | Users | 396 | 19.0 | 1,074 | 12.9 | 1.61 | 1.41–1.83 | 1.19^**^ | 1.02–1.38 |
| 50~59 | Nonusers | 3,410 | 77.1 | 14,845 | 83.9 | 1 | - | 1 | - |
|  | Users | 1,012 | 22.9 | 2,843 | 16.1 | 1.56 | 1.44–1.70 | 1.10^**^ | 1.00–1.21 |
| 60~69 | Nonusers | 5,094 | 72.5 | 22,336 | 79.4 | 1 | - | 1 | - |
|  | Users | 1,937 | 27.5 | 5,788 | 20.6 | 1.48 | 1.40–1.58 | 1.11^**^ | 1.04–1.19 |
| 70~79 | Nonusers | 6,443 | 68.0 | 28,582 | 75.4 | 1 | - | 1 | - |
|  | Users | 3,036 | 32.0 | 9,334 | 24.6 | 1.46 | 1.39–1.54 | 1.07^**^ | 1.01–1.13 |
| 80~89 | Nonusers | 3,818 | 66.4 | 17,351 | 75.5 | 1 | - | 1 | - |
|  | Users | 1,930 | 33.6 | 5,641 | 24.5 | 1.58 | 1.48–1.68 | 1.15^**^ | 1.07–1.24 |
| 90~99 | Nonusers | 627 | 71.4 | 2,805 | 79.9 | 1 | - | 1 | - |
|  | Users | 251 | 28.6 | 707 | 20.1 | 1.61 | 1.35–1.91 | 1.28^**^ | 1.05–1.56 |

* Adjusted for covariates presented in Table 1 (sex, age, underlying disease, Charlson comorbidity index, medication use, history of procedure/surgery)
** p < 0.05

† p-trend<0.001

**Table 3. Frequency of underlying disease and mediation use in exposed and unexposed controls**

|  | **Control**  **(N=118,552)** | | | |
| --- | --- | --- | --- | --- |
|  | **FQs users**  **(N=25,387)** | | **Nonusers**  **(N=93,165)** | |
|  | **N** | **%** | **N** | **%** |
| **Underlying disease** |  |  |  |  |
| Cerebrovascular disease | 2,051 | 8.1 | 4,367 | 4.7 |
| Arterial disease | 6,591 | 26 | 15,489 | 16.6 |
| Ischemic heart disease | 4,560 | 18 | 10,155 | 10.9 |
| Cardiac valve disease | 182 | 0.7 | 262 | 0.3 |
| Conduction disorder | 78 | 0.3 | 194 | 0.2 |
| Heart failure or cardiomyopathy | 1,454 | 5.7 | 2,654 | 2.8 |
| Chronic obstructive pulmonary disease | 10,219 | 40.3 | 21,507 | 23.1 |
| Pneumonia | 4,008 | 15.8 | 5,039 | 5.4 |
| Cancer | 2,844 | 11.2 | 5,618 | 6 |
| Liver disease | 9,686 | 38.2 | 22,326 | 24 |
| Renal disease | 1,061 | 4.2 | 1,841 | 2 |
| Rheumatism | 1,862 | 7.3 | 3,858 | 4.1 |
| Psychiatric disorder | 12,148 | 47.9 | 29,502 | 31.7 |
| Diabetes | 9,613 | 37.9 | 24,087 | 25.9 |
| Hypertension | 15,300 | 60.3 | 45,220 | 48.5 |
| Lipid disorder | 13,791 | 54.3 | 36,477 | 39.2 |
| Trauma | 15,136 | 59.6 | 38,144 | 40.9 |
| Obstructive sleep apnea | 55 | 0.2 | 135 | 0.1 |
| Asthma | 6,704 | 26.4 | 13,121 | 14.1 |
| Obesity | 26 | 0.1 | 90 | 0.1 |
| Seizure disorder | 959 | 3.8 | 2,056 | 2.2 |
| Decubitus ulcer | 9 | 0 | 17 | 0 |
| Infectious disease | 14,625 | 57.6 | 32,256 | 34.6 |
| Hypothyroidism | 1,653 | 6.5 | 3,434 | 3.7 |
| Inflammatory bowel disease | 3,894 | 15.3 | 7,879 | 8.5 |
| Urinary tract infection | 2,658 | 10.5 | 2,784 | 3 |
| Ehlers–Danlos syndrome | 0 | 0 | 0 | 0 |
| **Medication use** |  |  |  |  |
| ACEI | 575 | 2.3 | 1,599 | 1.7 |
| Antiarrhythmic | 10,657 | 42 | 23,507 | 25.2 |
| Anticonvulsant | 2,911 | 11.5 | 5,978 | 6.4 |
| Antidepressant | 4,968 | 19.6 | 9,098 | 9.8 |
| Immunodepressant | 9,873 | 38.9 | 17,347 | 18.6 |
| Anticoagulant | 19,764 | 77.9 | 48,963 | 52.6 |
| β-blocker | 4,648 | 18.3 | 10,725 | 11.5 |
| Oral hypoglycemic agent | 5,012 | 19.7 | 13,850 | 14.9 |
| Benzodiazepine | 9,763 | 38.5 | 20,920 | 22.5 |
| Calcium Channel Blockers | 10,032 | 39.5 | 28,184 | 30.3 |
| corticosteroid | 17,495 | 68.9 | 38,679 | 41.5 |
| DMARDS | 607 | 2.4 | 952 | 1 |
| INSULIN | 1,220 | 4.8 | 1,656 | 1.8 |
| Loop diuretics | 2,343 | 9.2 | 3,007 | 3.2 |
| NSAIDS | 21,985 | 86.6 | 60,519 | 65 |
| Antipsychotic | 7,749 | 30.5 | 15,512 | 16.7 |
| Peripheral vasodilators | 1,438 | 5.7 | 2,677 | 2.9 |
| Lipid-lowering agent | 8,504 | 33.5 | 22,498 | 24.1 |
| Parkinson medication | 1,835 | 7.2 | 4,385 | 4.7 |
| Hydroxyzine | 2,565 | 10.1 | 5,484 | 5.9 |

**Table 4. Association between AA/AD and FQ use in patients with cardiovascular diseases or indications of FQs**

| **Category** | | **Case** | | **Control** | | **Crude OR** | | **Adjusted OR*** | |
| --- | --- | --- | --- | --- | --- | --- | --- | --- | --- |
|  |  | **N** | **%** | **N** | **%** | **OR** | **95% CI** | **OR** | **95% CI** |
| Main analysis | Nonusers | 21,076 | 71.1 | 93,165 | 78.6 | 1 | - | 1 | - |
|  | Users | 8,562 | 28.9 | 25,387 | 21.4 | 1.51^**^ | 1.47–1.56 | 1.10^**^ | 1.07–1.14 |
| **Cardiovascular disease (risk factor of AA/AD)** | | | | | | | | | |
| Cardiovascular disease | Nonusers | 13,998 | 67.6 | 39,927 | 73.8 | 1 | - | 1 | - |
|  | Users | 6,720 | 32.4 | 14,207 | 26.2 | 1.42^**^ | 1.37–1.47 | 1.10^**^ | 1.06–1.15 |
| Arterial disease | Nonusers | 3,014 | 61.3 | 4,902 | 68.4 | 1 | - | 1 | - |
|  | Users | 1,903 | 38.7 | 2,265 | 31.6 | 1.40^**^ | 1.29–1.51 | 1.10^**^ | 1.00–1.20 |
| Hypertension | Nonusers | 12,291 | 67.7 | 32,563 | 74.0 | 1 | - | 1 | - |
|  | Users | 5,860 | 32.3 | 11,429 | 26.0 | 1.42^**^ | 1.36–1.48 | 1.08^**^ | 1.03–1.13 |
| **Indication of FQs** | | | | | | | | | |
| Pneumonia | Nonusers | 574 | 46.9 | 832 | 56.3 | 1 | - | 1 | - |
|  | Users | 651 | 53.1 | 646 | 43.7 | 1.55^**^ | 1.32–1.82 | 1.27^**^ | 1.06–1.52 |
| Infectious disease | Nonusers | 7,460 | 61.3 | 17,209 | 68.3 | 1 | - | 1 | - |
|  | Users | 4,706 | 38.7 | 8,003 | 31.7 | 1.38^**^ | 1.32–1.45 | 1.09^**^ | 1.04–1.15 |
| Urinary tract infection | Nonusers | 250 | 44.5 | 322 | 51.4 | 1 | - | 1 | - |
|  | Users | 312 | 55.5 | 304 | 48.6 | 1.36^**^ | 1.07–1.72 | 1.05 | 0.79–1.38 |

* Adjusted for covariates presented in Table 1 (sex, age, underlying disease, Charlson comorbidity index, medication use, history of procedure/surgery)
** p < 0.05
